# Long-term oncological outcomes after local excision of T1 rectal cancer

**DOI:** 10.1007/s10151-022-02661-6

**Published:** 2022-08-27

**Authors:** J. W. A. Leijtens, L. J. H. Smits, T. W. A. Koedam, R. G. Orsini, S. M. van Aalten, M. Verseveld, P. G. Doornebosch, E. J. R. de Graaf, J. B. Tuynman

**Affiliations:** 1 Department of Surgery, Laurentius Hospital, Roermond, The Netherlands; 2 Department of Surgery, Amsterdam UMC, Vrije Universiteit Amsterdam, Cancer Centre Amsterdam, Amsterdam, The Netherlands; 3 Department of Surgery, Elisabeth-Tweesteden Ziekenhuis, Tilburg, The Netherlands; 4 Department of Surgery, Groene Hart Ziekenhuis, Gouda, The Netherlands; 5 Department of Surgery, Franciscus Gasthuis & Vlietland, Rotterdam/Schiedam, The Netherlands; 6 Department of Surgery, IJsselland Ziekenhuis, Capelle Aan Den IJssel, The Netherlands

**Keywords:** Rectal cancer, Early cancer, Surgical local excision, Oncological outcome

## Abstract

**Background:**

A growing proportion of patients with early rectal cancer is treated by local excision only. The aim of this study was to evaluate long-term oncological outcomes and the impact of local recurrence on overall survival for surgical local excision in pT1 rectal cancer.

**Methods:**

Patients who only underwent local excision for pT1 rectal cancer between 1997 and 2014 in two Dutch tertiary referral hospitals were included in this retrospective cohort study. The primary outcome was the local recurrence rate. Secondary outcomes were distant recurrence, overall survival and the impact of local recurrence on overall survival.

**Results:**

A total of 150 patients (mean age 68.5 ± 10.7 years, 57.3% males) were included in the study. Median length of follow-up was 58.9 months (range 6–176 months). Local recurrence occurred in 22.7% (*n* = 34) of the patients, with a median time to local recurrence of 11.1 months (range 2.3–82.6 months). The vast majority of local recurrences were located in the lumen. Five-year overall survival was 82.0%, and landmark analyses showed that local recurrence significantly impacted overall survival at 6 and 36 months of follow-up (6 months, *p* = 0.034, 36 months, *p* = 0.036).

**Conclusions:**

Local recurrence rates after local excision of early rectal cancer can be substantial and may impact overall survival. Therefore, clinical decision-making should be based on patient- and tumour characteristics and should incorporate patient preferences.

**Supplementary Information:**

The online version contains supplementary material available at 10.1007/s10151-022-02661-6.

## Introduction

Currently, the standard treatment for rectal cancer is total mesorectal excision (TME). TME surgery may be combined with (chemo)radiotherapy according to the stage of disease [1]. This approach provides a good oncological outcome, but leads to morbidity, affects functional outcome, and influences quality of life [2–4]. To avoid the negative consequences of TME surgery there has been a rising interest in local treatment regimens, especially in early rectal cancer.

In the earliest stage of rectal cancer, local excision by surgical or endoscopic techniques has become the treatment of choice [5]. If the histopathological evaluation of the excised specimen shows risk factors for nodal disease and/or recurrence, completion TME is recommended by guidelines [5]. Nevertheless, this advice is frequently waived by surgeons and/or patients [6]. During the clinical- and shared decision-making process, morbidity and expected functional outcome are weighed against the chance of local recurrence. The risk of local recurrence reported in the literature is highly variable and randomised trials are lacking. A recent meta-analysis of available cohort studies with at least 36 months of follow-up, reported an overall local recurrence rate of 8.1% after local excision of T1 rectal cancer [7]. However, local recurrence rates of the included studies varied from 0 to 40% [7].

Since the clinical consequences of treatment strategies (i.e. organ preservation or radical surgery) are substantial, shared decision-making should be based upon reliable data. In addition, the feasibility of salvage surgery and the impact of local recurrence on overall survival (OS) should also be considered. Therefore, the aim of this study was to evaluate the long-term oncological outcomes of surgical local excision techniques for pT1 rectal cancer. The primary outcome was local recurrence rate. Secondary outcomes were distant recurrence rate, overall survival (OS) rate, and the impact of local recurrence on survival.

## Materials and methods

### Patient selection

Between 1997 and 2014 all patients who only underwent surgical local excision of pT1 rectal cancer, in two Dutch tertiary referral hospitals for local excision (Laurentius Hospital and IJsselland Hospital) were included. Local excision was performed by either transanal endoscopic microsurgery (TEM) or transanal minimally invasive surgery (TAMIS), and consisted of a full thickness excision. Surgical local excision was performed in patients with lesions < 4 cm or if the invasive part of the lesion was estimated to be < 3 cm based on endoscopy. Local excision was also performed in patients who were staged beyond cT1, because of the known inaccuracy of imaging in early staged rectal cancer [8]. Nevertheless, all patients had histopathologically proven pT1 rectal cancer, defined as a tumour within 15 cm from the anal verge. Patients were excluded in case of: conversion to TME; suspected lymph node involvement at clinical staging; or distant metastases at the time of diagnosis. Clinical staging was performed in all patients and was based on imaging by endorectal ultrasound (ERUS), abdominopelvic computed tomography (CT) scan, and chest X-ray or CT scan. Pelvic magnetic resonance imaging (MRI) was implemented as well during the study period. Several reasons for local excision as a sole treatment were applicable. The included patients either did not require completion TME according to the national guidelines, were clinically unfit to undergo completion surgery, or waived additional treatment by TME surgery during a shared decision-making process [9]. Patients who underwent completion TME were excluded. The study was approved by the Institutional Review Boards of both participating centres.

### Surgical procedures and histopathological examination

Excisions were performed according to the TEM or TAMIS technique, as described by Buess et al. and Atallah et al. [10, 11]. A full thickness resection was performed by experienced surgeons. After resection, the specimen was oriented, pinned on cork, fixed in formaldehyde, and sent for histopathological examination. Histopathological assessment included lesion size (i.e. polyp size including tumour), pT-stage, submucosal invasion depth (i.e. Kikuchi classification for sessile lesions), differentiation grade, lymphovascular invasion, and residual tumour classification (R-classification). Tumour budding was not included in the histopathological evaluation, since this was not incorporated in the standardised histopathology reports.

### Data collection and follow-up

Patient- and tumour characteristics, as well as perioperative data, were collected. Postoperative complications were scored according to the Clavien-Dindo classification [12]. Based on histopathological tumour characteristics patients were divided into low-risk pT1 tumours and high-risk pT1 tumours. In high-risk tumours at least one of the following characteristics had to be present: poor differentiation, lymphovascular invasion, involved resection margin ≤ 1 mm (residual tumour classification R1), or either unclear or not evaluable resection margins (Rx). In low-risk tumours all these factors had to be absent. Guidelines are conflicting with regard to deep submucosal invasion as a risk factor. Moreover, a recent meta-analysis reported that deep submucosal invasion (i.e. ≥ 1000 µm or Kikuchi level sm2-3) is not an independent risk factor for lymph node metastasis [13]. Therefore, deep submucosal invasion was not included as a high-risk factor.

Follow-up included visits to the outpatient clinic, physical examinations, abdominal ultrasound, chest X-rays and measurements of carcinoembryonic antigen (CEA) levels every 6 months for 3 years, and annually thereafter until 5 years of follow-up. Twelve months after local excision a colonoscopy was performed. The majority of patients received additional periodical surveillance, which included MRI and/or sigmoidoscopies with or without ERUS, every 3 months for the first year after local excision in low-risk pT1 tumours, and during the first 2 years in high-risk pT1 tumours. These patients were defined as the close surveillance group.

The primary study objective was local recurrence, which was defined as pelvic recurrence, and could either be endoluminal located at the local excision scar; or locoregional lymph node recurrence. Preferably, local recurrence was confirmed by a biopsy, if a biopsy could not be obtained, the diagnosis was based on imaging (i.e. MRI/CT/ERUS). Secondary outcomes were distant recurrence, eligibility for salvage surgery in case of local recurrence, local recurrence free survival, OS, and the impact of local recurrence on OS.

### Statistical analysis

Patient characteristics, perioperative data and clinical outcomes were evaluated using descriptive statistics. Categorical data were presented by frequencies and percentages. Continuous data were differentiated into variables with a normal distribution and a non-normal distribution based on Q-Q plots. Normally distributed variables were reported as mean with standard deviation and non-normally distributed data by median and ranges. Kaplan–Meier curves and Log Rank tests were performed to investigate associations between categorical variables and time to local recurrence. For continuous variables Cox regression analyses were performed after testing the proportional hazards assumption by Schoenfeld residuals. A similar method was used to assess the influence of non-time dependent variables on local recurrence free survival and OS. Kaplan–Meier curves were used to estimate local recurrence free survival rates and OS rates. Since local recurrence is a time-dependent variable, the influence of local recurrence on OS was analysed in depth using landmark analyses [14]. In a landmark analysis the influence of local recurrence on OS is estimated at a certain time point, analysing only the patients who have survived until this time point [15]. During the analysis OS rates are estimated for patients with- or without recurrence at the set landmark time. If patients are simply categorised by the presence of recurrence during the entire length of follow-up, the timing of recurrence and length of survival itself will create a bias. Therefore, landmark analyses provide a less biased estimation of the time-to-event outcome local recurrence [16, 17]. Landmarks were set on 6, 12, 24, and 36 months of follow-up. *P-*values of < 0.05 were considered statistically significant. Statistical analyses were carried out using SPSS version 26 (IBM Corp., Armonk, NY, USA).

## Results

### Patient characteristics and clinical outcomes

From January 1997 to September 2014 a total of 150 patients underwent solely surgical local excision for pT1 rectal cancer. Their mean age was 68.5 ± 10.7 years, and 57.3% were men (Table 1). Table 1 shows peri- and postoperative outcomes. The majority of patients were clinically staged as benign or cT1. One patient staged as cT3, had severe comorbidities and was deemed unfit for TME surgery. Four cT2 patients opted for local excision to have a chance of organ preservation. Median duration of the surgical procedure was 50.0 min (range 10–245 min). Intra-operative complications occurred in 2.0% (*n* = 3) of the patients and consisted of arterial bleeding (*n* = 2) or perforation with peritoneal breach (*n* = 1). Perforation occurred in a patient who underwent a polypectomy prior to the local excision. The specimen did not show residual tumour. In total 4.6% (*n* = 7) of the patients had a postoperative complication and 2.6% (*n* = 4) scored grade III or higher according to the Clavien-Dindo classification. No procedure related deaths were recorded. Seventy-eight percent of the patients (*n* = 117) underwent close surveillance, others underwent regular follow-up.

**Table 1:** Demographic, clinical and tumour characteristics (*n* = 150)

	*n* (%)
Sex; Male	86 (57.3)
Age in years at time of surgery, mean ± SD	68.5 ± 10.7
Follow-up in months, median (range)	58.9 (6–176)
BMI in kg/m^2^, mean ± SD	26.2 ± 3.8
ASA classification	
I-II	129 (86.0)
III	18 (12.0)
Missing	3 (2.0)
Distance in cm from anal verge	
0–5	51 (34.0)
6–10	69 (46.0)
11–15	25 (16.7)
Missing	5 (3.3)
Location of tumour	
Anterior	43 (28.7)
Posterior	46 (30.7)
Lateral right	17 (11.3)
Lateral left	28 (18.7)
Missing	16 (10.7)
Preoperative staging	
Benign	34 (22.7)
cT1	58 (38.7)
cT2	4 (2.7)
cT3	1 (0.7)
Missing	53 (35.3)
Duration of surgical procedure in minutes, median (range)	50 (10–245)
Type of procedure	
TEM	127 (84.7)
TAMIS	18 (12.0)
Missing	5 (3.3)
Closure of defect	
No	4 (2.7)
Yes	130 (86.7)
Missing	16 (10.7)
Peroperative complication	3 (2.0)
Length of stay in days, median (range)	3 (1–24)
Complications according to Clavien-Dindo	
No complications	143 (95.3)
Grade I–II	3 (2.0)
Grade IIIb	2 (1.3)
Grade IVb	2 (1.3)
Radicality of resection	
R0	141 (94.0)
R1/Rx	9 (6.0)
Pathological size of lesion in cm	
Scar	25 (16.7)
< 3	49 (32.7)
≥ 3	56 (37.3)
Missing	20 (13.3)
Grade of differentation	
Well-to-moderate	126 (84.0)
Poor	7 (4.7)
Missing	17 (11.3)
Lymphovascular invasion	
No	106 (70.7)
Yes	14 (9.3)
Missing	30 (20.0)
Kikuchi classifcation	
sm1	50 (33.3)
sm2	6 (4.0)
sm3	17 (11.3)
Missing	77 (51.3)
Risk of tumour^a^	
Low-risk	93 (62.0)
High-risk	29 (19.3)
Unknown	28 (18.7)

*BMI* body mass index, *ASA* American Society of Anesthesiologists, *TEM* transanal endoscopic microsurgery, *TAMIS* transanal minimally invasive surgery

^a^High-risk defined as poor differentiation, lymphovascular invasion, distance to resection plane ≤ 1 mm or unevaluable

### Histological examination

Ninety-four percent (*n* = 141) of the patients had clear resection margins (R0) after surgical local excision. No association between resection margins and local recurrence was observed. Tumours were poorly differentiated in 4.7% (*n* = 7) of the patients. Lymphovascular invasion was present in 9.3% (*n* = 14) of the patients. Table 1 provides an overview of histopathological risk factors. Combining histopathological risk factors resulted in 19.2% (*n* = 29) of the patients with a high-risk pT1 tumour. In 1 case, analysis of the specimen revealed a lymph node with tumour invasion.

### Oncological outcomes

Median follow-up of all patients was 58.9 months (range 6–175 months). Local recurrence occurred in 22.7% (*n* = 34) of the 150 patients, with a median time to local recurrence of 11.1 months (range 2.3–82.6 months) (Table 2). Of these local recurrences, preoperative assessment by endoscopy and imaging revealed that 85.3% (*n* = 29) of the recurrences were located endoluminally at the scar of the local excision, and 14.7% (*n* = 5) patients had suspected mesorectal lymph node recurrences. Distant recurrence was reported in 7.3% (*n* = 11) of the patients. Of the 11 patients with distant recurrence, 81.8% (*n* = 9) had both local and distant recurrences. No significant associations between patient- and tumour characteristics, such as lesion size, and local recurrence rate were found, except for the presence of lymphovascular invasion (*p* = 0.017, Table 3). Local recurrence was associated with the occurrence of distant metastases (Fisher’s exact test, *p* < 0.001). Table 2 shows the characteristics of recurrences.

**Table 2:** Recurrences of pT1 rectal cancer patients after TEM/TAMIS (*n* = 36)

	*n* (%)
Tumour risk	
Low-risk	24 (66.7)
High-risk	9 (25.0)
Missing	3 (8.3)
Location recurrence	
Local	34 (94.4)
Distant	11 (30.6)
Local and distant	9 (25.0)
Median time to local recurrence in months	11.1 (2.3–82.6)
Median time from TEM/TAMIS to salvage surgery in months	12.5 (4.4–58.4)
Location of local recurrence (pre-operative assessment)	
Intraluminal	29 (85.3)
Nodal recurrence	5 (14.7)
Treatment of local recurrence	
No surgery	7 (20.6)
Salvage surgery	27 (79.4)
APR	13 (48.1)
LAR	14 (51.9)
Radiotherapy	
None/not applicable	9 (26.5)
Short-course radiotherapy	13 (38.2)
Chemoradiation	11 (32.4)
Long course radiotherapy	1 (2.9)
Pathological T-stage local recurrence	
pT0	2 (7.4)
pT1	5 (18.5)
pT2	2 (7.4)
pT3	12 (44.4)
pT4	2 (7.4)
Missing	4 (14.8)
Pathological N-stage local recurrence	
pN0	12 (44.4)
pN1	9 (33.3)
pN2	2 (7.4)
Missing	4 (14.8)
Local recurrence diagnosed based on	
MRI, endoscopy, ERUS	14 (41.2)
MRI, endoscopy	5 (14.7)
MRI, ERUS	1 (2.9)
Endoscopy, ERUS	1 (2.9)
MRI	7 (20.6)
Endoscopy	5 (14.7)
Missing	1 (2.9)

*TEM* transanal endoscopic microsurgery, *TAMIS* transanal minimally invasive surgery, *APR* abdominal perineal resection, *LAR* low anterior resection, *MRI* magnetic resonance imaging, *ERUS* endorectal ultrasound

**Table 3:** Factors associated with 5-year local recurrence by Kaplan Meier estimates

	No local recurrence *n* = 117 (%)	Local recurrence *n* = 33 (%)	*p* *v*alue
Age in years, mean ± SD^a^	68.8 ± 11.0	67.6 ± 9.7	0.75
Sex			0.21
Male	70 (81.4)	16 (18.6)	
ASA classification			0.86
ASA I-II	101 (78.3)	28 (21.7)	
ASA III	14 (77.8)	4 (22.2)	
Distance from anal verge in cm			0.98
0–5	39 (76.5)	12 (23.5)	
6–10	53 (76.8)	16 (23.2)	
11–15	20 (80.0)	5 (20.0)	
Location of tumour			0.43
Anterior	35 (81.4)	8 (18.6)	
Posterior	33 (71.7)	13 (28.3)	
Lateral right	15 (88.2)	2 (11.8)	
Lateral left	21 (75.0)	7 (25.0)	
Type of procedure			0.96
TEM	100 (78.7)	27 (21.3)	
TAMIS	14 (77.8)	4 (22.2)	
Duration of surgical procedure in minutes			0.057
0–59	71 (84.5)	13 (15.5)	
60–89	28 (71.8)	11 (28.2)	
≥ 90	10 (62.5)	6 (37.5)	
Peroperative complication			0.37
Yes	3 (10)	0 (0)	
Postoperative complications according to Clavien- Dindo			0.58
Grade I–II	3 (100)	0 (0)	
Grade IIIb	2 (100)	0 (0)	
Grade IVb	1 (50)	1 (50)	
Radicality of resection			0.46
R1/R*x*	8 (88.9)	1 (11.1)	
Pathological size of lesion in cm			0.53
Scar	21 (84.0)	4 (16.0)	
< 3	38 (77.6)	11 (22.4)	
≥ 3	40 (71.4)	16 (28.6)	
Grade of differentiation			0.58
Well-to-moderate	96 (76.2)	30 (23.8)	
Poor	6 (85.7)	1 (14.3)	
Lymphovascular invasion			0.017
Yes	7 (50)	7 (50)	
Risk of tumour^b^			0.39
Low-risk	71 (76.3)	22 (23.7)	
High-risk	20 (69.0)	9 (31.0)	
Year of procedure, median (range)	2007 (1997–2014)	2007 (1999–2013)	

*ASA* American Society of Anesthesiologists, *TEM* transanal endoscopic microsurgery, *TAMIS* transanal minimally invasive surgery

^a^Cox regression analysis

^b^High-risk defined as poor differentiation, lymphovascular invasion, distance to resection plane ≤ 1 mm or unevaluable

Salvage surgery was performed in 79.4% (*n* = 27) of the patients with local recurrence, and consisted of a low anterior resection in 51.9% (*n* = 14), and an abdominoperineal resection in 48.1% (*n* = 13) of the patients. Postoperative complications occurred in 33.3% (*n* = 9) of the patients. According to the Clavien-Dindo classification 5 patients had grade II and 4 patients had grade III complications. Prior to the salvage procedure 73.5% (*n* = 20) received neoadjuvant (chemo) radiotherapy. Outcomes of the histopathological evaluation of the salvage specimen could be retrieved in 85.2% (*n* = 23) of the patients, and revealed that 51.9% (*n* = 14) of the patients had a pT3–4 tumour, and 40.7% (*n* = 11) had tumour positive lymph nodes (i.e. pN1-2) (Table 2). In 1 patient pathological assessment of the specimen showed complete response after neoadjuvant treatment. Median length of follow-up after salvage surgery was 30.5 months (range 7–111 months). Of the 27 patients that underwent salvage surgery, 14.8% (*n* = 4) eventually died due to disease progression. Close surveillance was not associated with a higher probability of salvage surgery (Fisher’s exact test, *p* = 0.28).

### Local recurrence free survival

**Fig. 1:**
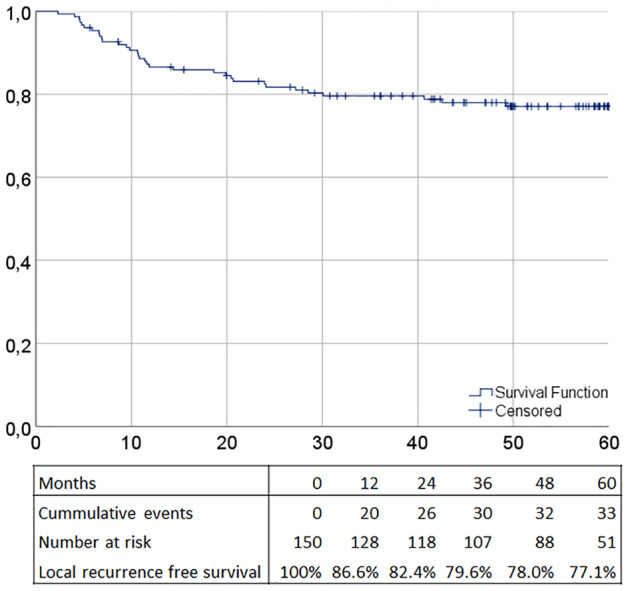
Kaplan–Meier curve of 60-month local recurrence free survival

Local recurrence free survival was 81.0% at 3 years and 77.1% at 5 years of follow-up (Table 4). The Kaplan–Meier curve for local recurrence free survival is provided in Fig. 1. No differences were observed in local recurrence free survival between low-risk tumours and high-risk tumours (low-risk 75.2% and high-risk 67.8%, HR = 1.41, 95% CI 0.65–3.06, *p* = 0.39) (Table 4).

**Table 4:** Survival outcomes

	3 year %	5 year %	5 year HR	95% CI	*p* value
Local recurrence free survival					
Overall	81.0	77.1			
Low-risk pT1	76.7	75.2	Ref	Ref	Ref
High-risk pT1	71.5	67.8	1.41	0.65–3.06	0.39
Overall survival					
Overall	91.1	82.0			
Low-risk pT1	93.4	82.7	Ref	Ref	Ref
High-risk pT1	89.4	75.0	1.51	0.58–3.99	0.40
Patients without local recurrence	93.1	85.1	Ref	Ref	Ref
Patients with local recurrence	84.1	67.7	2.41	1.05–5.50	0.037
Recurrences					
Normal surveillance with recurrence	80.0	80.0	Ref	Ref	Ref
Close surveillance with recurrence	69.4	63.1	1.94	0.24–15.58	0.53

### OS

**Fig. 2:**
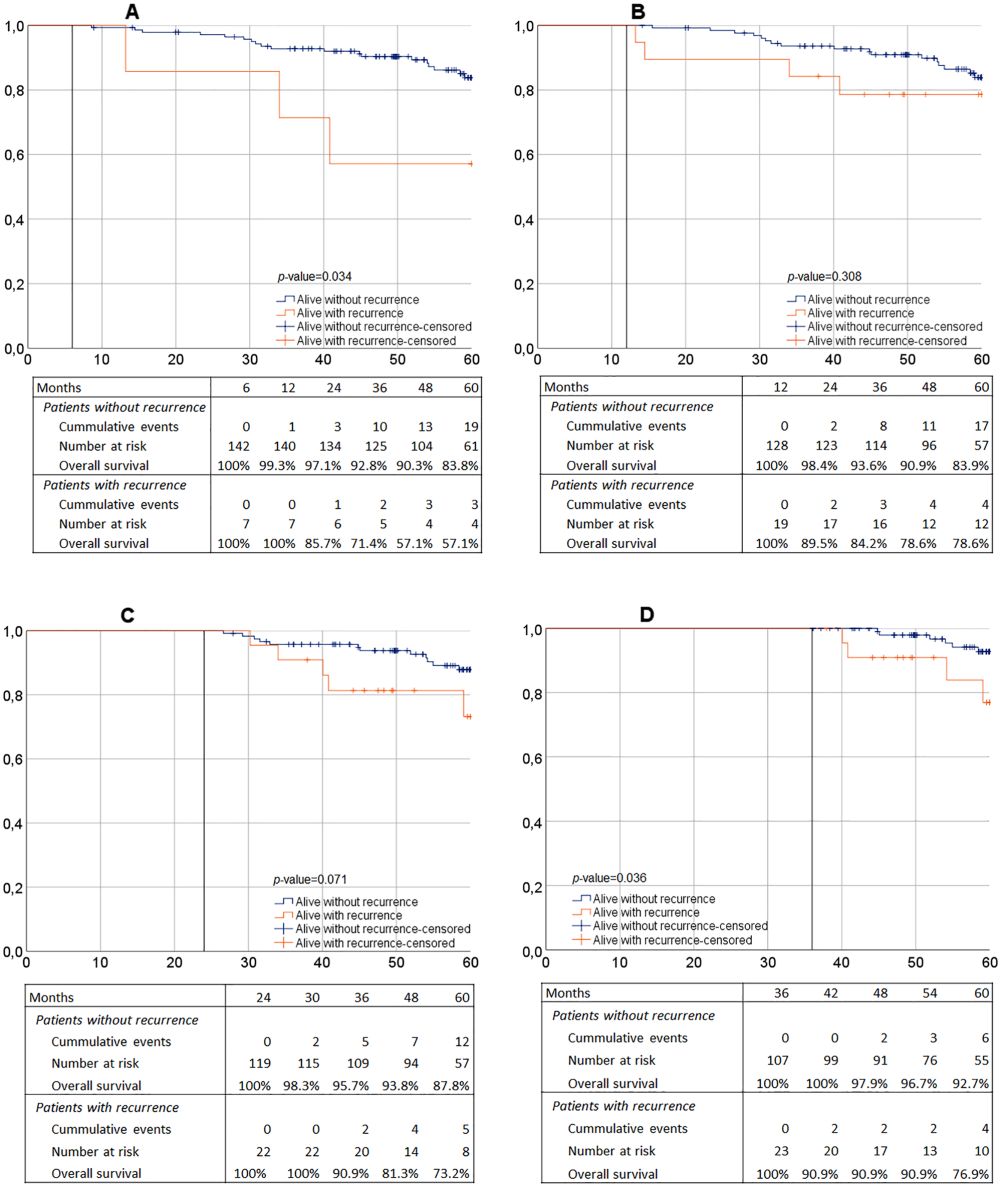
Landmark analyses. **A** 6-month landmark. **B** 12-month landmark. **C** 24-month landmark. **D** 36-month landmark. In depth analyses of overall survival based on patients alive at the set landmark. The curves provide estimates for patients with- or without recurrence at the set landmark time

OS was 91.1 and 82.0% at 3 and 5 years of follow-up, respectively (Table 4, Supplementary Fig. 1). No significant associations between patient- and tumour characteristics and OS were found except for age (HR = 1.10 95% CI 1.05–1.15, *p* < 0.001). No differences in OS rates were observed between low- and high-risk pT1 tumours (Table 4). Moreover, in patients with local recurrence no differences in OS rates could be detected for normal follow-up or close surveillance (Table 4). Kaplan–Meier estimates suggested that patients with local recurrence had a worse 5-year OS compared to patients without local recurrence (67.6 vs. 86.0% respectively, *p* = 0.023, Supplementary Fig. 2). In depth landmark analyses showed that for patients with local recurrence at 6 and 36 months, OS was significantly lower (6 months: *p* = 0.034, 36 months: *p* = 0.036, Fig. 2). Even though the 24 months landmark showed a trend towards lower OS as well, similar outcomes could not be determined for the landmark of 12 months of follow-up (12 months: *p* = 0.31, 24 months: *p* = 0.071, Fig. 2).

## Discussion

In this cohort study of 150 patients with only locally excised pT1 rectal cancers there was a high local recurrence rate of 22.7%, with a median time to local recurrence of approximately 11 months. The majority of the local recurrences were detected endoluminally, local recurrence was associated with the occurrence of distant metastases, and approximately 80% of recurrences were eligible for salvage surgery. In addition, our results suggest that local recurrences may negatively impact OS.

After local excision of high-risk pT1 tumours, radical surgery is recommended to reduce the risk of local recurrence [1, 18]. A recent meta-analysis showed that overall local recurrence rates after local excision of high-risk pT1 tumours can be estimated to be 14% [7]. This conclusion is hampered by a lack of large cohort studies or clinically controlled trials and selection- and/or publication bias of the included cohort studies. It has been suggested, that the local recurrence rate may be slightly higher after surgical local excision compared with endoscopic, however, other studies do not support this outcome and high-quality evidence is lacking [19–21]. Nevertheless, the overall local recurrence rate of 22.7% in the current study is substantial, in particular since the majority of investigated tumours were low-risk tumours and surgery was performed by surgeons experienced in local excision. There may be several explanations for the local recurrence rate. First, due to incomplete reports, an underestimation of histopathological risk factors might be present in this cohort, which may be emphasized by the fact that we were unable to identify an association between high-risk tumours and local recurrence. Moreover, previous studies have shown that interobserver variability of histopathological risk factors is high, and over the past years the increasing evidence about the importance of identification of risk factors in early cancers has led to more detailed histopathology reports [22–24]. Another possible explanation might be the quality of the resected specimens. Nonetheless, 94.0% of the resections were complete (R0), and R-classification was not associated with local recurrence. In addition to involved or unevaluable resection margins, alternative hypotheses for the development of local recurrence, such as the influence of tissue handling, have been proposed [25]. The importance of avoiding fragmented resections seems evident, however, the role of tumour seeding might be underestimated [25, 26]. Tumour seeding theories propose that inadequate handling of tissue and instruments cause implantation of viable tumour cells into the damaged mucosa, which leads to local recurrence at the site of the excision [27]. These hypotheses do not agree with the general idea that recurrences after local excision mainly consist of unidentified lymph node involvement. The reported trend towards an association between the duration of the procedure and local recurrence might suggest a link between difficult resections and therefore tissue handling, and local recurrence. Moreover, the high proportion of endoluminal recurrences supports this theory. Although short-term outcomes of local excision techniques have been studied thoroughly, there seems to be room for further investigation into the influence of procedure related factors on local recurrence. Next to specimen quality and histopathological characteristics, surgical quality of the procedure including potential tumour spill might be an important risk factor for local recurrence. Future research is needed to address these factors and potentially improve oncological outcomes.

Approximately 80% of the patients with local recurrence were eligible for salvage surgery. In a small number of studies the percentage of patients eligible for salvage surgery in case of local recurrence was investigated and varied from 73 to 92%, which corresponds to our findings [28–32]. Few studies have investigated eligibility and outcomes of salvage surgery in recurrences after local excision. To establish accurate outcomes of patients with local recurrence after local excision of early rectal cancer, more robust data is necessary. Based on the limited evidence available, cancer specific and overall survival seems poor. Two smaller cohort studies reported 3- and 5- year cancer free survival of 58% and 53%, respectively. A systematic review by Jones and colleagues, described an overall survival rate of 50% after salvage treatment, conceivably due to the occurrence of distant metastases [33]. In adverse outcomes of early tumours, there may be an important role for tumour biology. However, it remains hard to identify and target these aggressive early tumours appropriately.

Even though this study reported a relatively high recurrence rate, the majority of patients with pT1 tumours will not get a recurrence, and local excision techniques are sufficient treatment for these patients. For this reason, the potential value of rectal preservation in early rectal cancer should be kept in mind. Treatment related morbidity and functional outcomes are of great importance to patients with rectal cancer [34, 35]. In addition, the trade-off between the risk of local recurrence and good functional outcomes may differ for each patient. These trade-offs cause patients and physicians to disregard recommendations of completion surgery. To provide patient tailored treatment, local excision followed by an assessment of risk factors seems appealing. In high-risk tumours additional treatment by completion surgery remains the standard. In particular given the reported high recurrence rate and the potential impact on survival in a patient population predominantly fit for additional treatment. A promising future perspective might be adjuvant chemoradiotherapy, which may provide better functional outcomes and less impact on quality of life compared with completion surgery, while maintaining an acceptable low risk of recurrence. Recently, a meta-analysis reported similar recurrence rates for adjuvant (chemo)radiotherapy and completion surgery in high-risk pT1 tumours. However, the current evidence on this treatment strategy is scarce and hampered by selection bias, therefore more long-term outcome data are necessary prior to implementation of this treatment strategy [7, 36].

One of the limitations of this study is caused by the condition of the included patients, which may have influenced overall survival. Although the majority of patients did not require completion surgery according to the guidelines, the patients with high-risk tumours either waived radical surgery or were clinically unfit to undergo it. However, at the time of the local excision, only 12 patients had a severe systemic disease, i.e. were American Society of Anesthesiologists (ASA) class III. Furthermore, due to the retrospective nature of the study some information could not be retrieved from the patient records. For example, in almost 20% of the cases we were unable to categorize the patient as having a low- or high-risk pT1 tumour, because risk factors were not reported in the histopathological report. Over time we observed a decrease in incomplete reports. Nonetheless, due to incomplete histopathological reports and known interobserver variability the true percentage of high-risk tumours may have been higher [22, 24]. In spite of the relatively high recurrence rate, only 34 patients had a local recurrence. The relatively small number of local recurrences may have caused some of the analyses to not reach statistical significance. Nevertheless, the current cohort is one of the largest cohorts that reports long-term outcomes of surgical local excision in pT1 tumours and counterbalances the optimistic outcomes of other studies [7].

## Conclusions

After solely surgical local excision for pT1 rectal cancer, local recurrences were observed in 1 out of 5 patients. Salvage surgery was possible in 80% of these patients, but local recurrence may impact survival negatively. Therefore, this study does not support the optimistic outcomes of earlier studies and shows that close surveillance is necessary to diagnose and treat local recurrence in an early stage. However, for the majority of patients with early rectal cancer local excision will be sufficient, consequently this strategy may be an option in selected patients. Selection of these patients should be based on patient- and tumour characteristics and during the shared decision-making process patients should be informed of the risks associated with local recurrence.

## Supplementary Information

Below is the link to the electronic supplementary material.

Supplementary figure 1 Kaplan-Meier curve of 60-month overall survival Supplementary file2 (TIF 5012 kb)

Supplementary figure 2 Kaplan-Meier curve, the influence of local recurrence on 60-month overall survival Supplementary file3 (TIF 5987 kb)
