# Supplementary figures and images for: Long-term oncological outcomes after local excision of T1 rectal cancer
Source: Tech Coloproctol. 2022 Aug 27;27(1):23–33. doi: 10.1007/s10151-022-02661-6 (PMC9807482; doi:10.1007/s10151-022-02661-6)

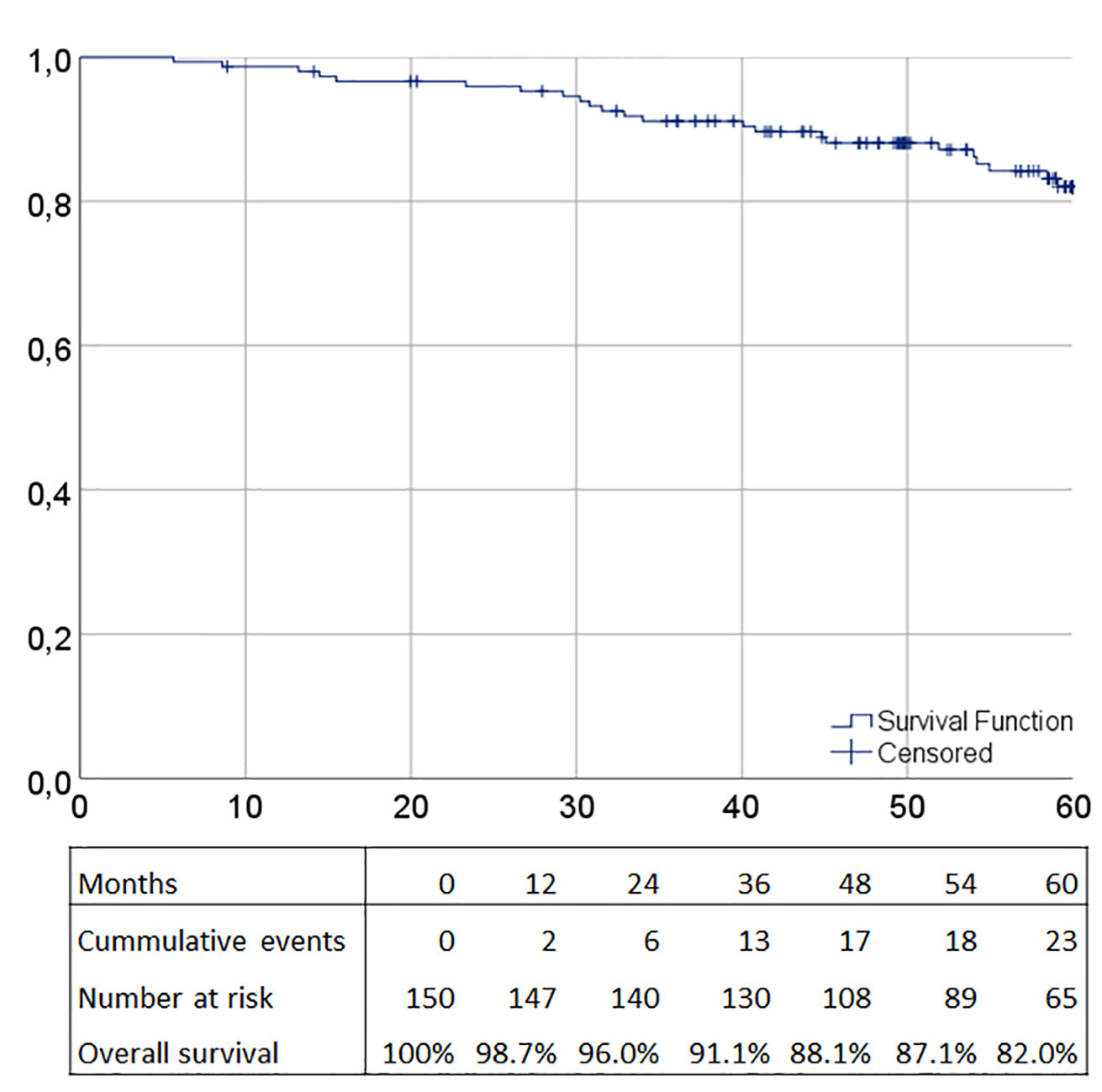

Supplement: Supplementary file 1 — Supplementary figure 1 Kaplan-Meier curve of 60-month overall survival Supplementary file2 (TIF 5012 kb) [file 10151_2022_2661_MOESM1_ESM.tif]

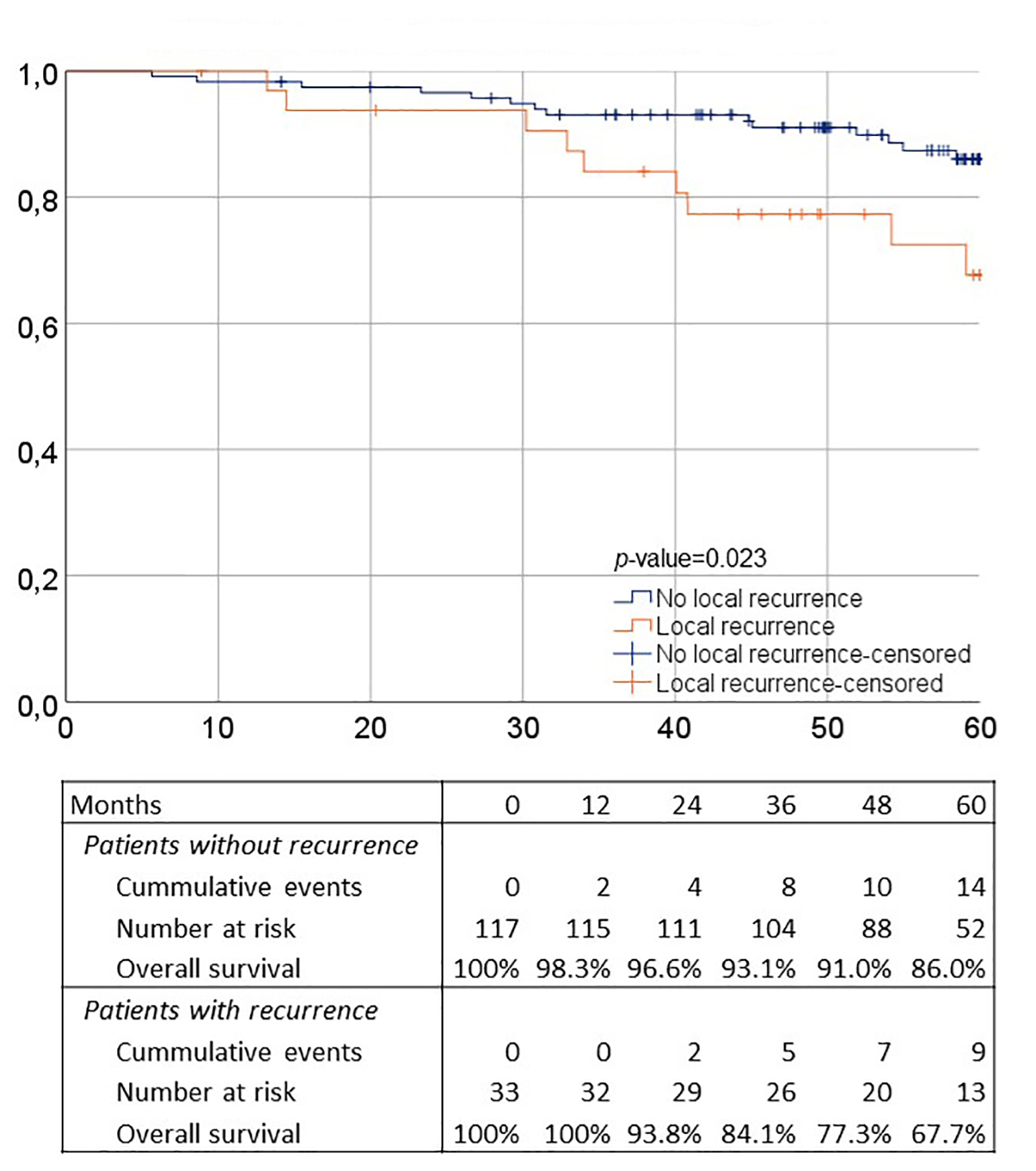

Supplement: Supplementary file 2 — Supplementary figure 2 Kaplan-Meier curve, the influence of local recurrence on 60-month overall survival Supplementary file3 (TIF 5987 kb) [file 10151_2022_2661_MOESM2_ESM.tif]
